# Potential novel biomarkers in small intestine for obesity/obesity resistance revealed by multi-omics analysis

**DOI:** 10.1186/s12944-022-01711-0

**Published:** 2022-10-08

**Authors:** Yueshan Pang, Yali Zheng, Ni Yang, Meng Zan, Lu Zhang, WeiJun Ding

**Affiliations:** 1 Department of Fundamental Medicine, Chengdu University of Traditional Chinese Medicine, 611130 Chengdu, China; 2 The Second Clinical Medical College, North SiChuan Medical College, 637000 Nanchong, China; 3 School of Pharmacy, Chengdu University of Traditional Chinese Medicine, 611130 Chengdu, China

**Keywords:** Obesity, Obesity resistance, High-fat diet, Gut microbiota, Cxcl10, 5-HT

## Abstract

**Background:**

Although obesity is caused by different factors, individual susceptibility to obesity differs among people under the same circumstances. The microbiota in the caecum or fresh faeces and metabolites in blood or urine contribute to obesity resistance; however, the microbiota or metabolites in the small intestine have not been extensively studied.

**Methods:**

To investigate the relationship between the microbiota or metabolites in the small intestine and susceptibility to obesity, eighty-eight male C57BL/6 mice were fed a high-fat diet (HFD) for 8 weeks to establish two models of obesity and obesity resistance. For further study, six mice were chosen from among the obesity models, and twelve mice were randomly chosen from among the obesity resistance models. After fasting plasma glucose and behavioural testing, the mice were fed in single cages for another 4 weeks to observe their weight and food intake. All mice were sacrificed at 20 weeks of age. Serum ALT, AST, HDL, LDL, TG and TC levels were measured using an automatic biochemical analyser. The microbiota and metabolites in the small intestine contents were analysed using 16 S sequencing and an ultrahigh-performance liquid chromatographic system, respectively. Transcripts in the jejunum were evaluated using full-length transcriptome sequencing and verified by qPCR.

**Results:**

The results showed that HFD induced depression and anxiety behaviours and higher fasting plasma glucose, ALT, AST, HDL, LDL, TG and TC levels in the obese mice; however, these levels were improved in obese resistance mice. The correlation analysis showed that the phosphatidylcholine, TG, and phosphatidylethanolamine levels were higher in obese mice and correlated positively with intestinal microflora (*Desulfovibrio* and *Gemella*) and the Cxcl10 gene. A higher abundance of *Clostridium_sensu_stricto_1* in obesity-resistant mice correlated negatively with the metabolite contents (neuromedin N and enkephalin L) and Pck1 gene expression and correlated positively with certain metabolites (5-hydroxy-L-tryptophan, cinnamyl alcohol and 1 H-indole-3-acetamide) and genes expression (Gdf15, Igfbp6 and Spp1).

**Conclusion:**

*Clostridium_sensu_stricto_1*, neuromedin N, enkephalin L, Pck1, 5-hydroxy-L-tryptophan, Cxcl10 and cinnamyl alcohol may be novel biomarkers in the small intestine for obesity/obesity resistance. These might be helpful for obesity prevention or for treating obese patients.

**Supplementary Information:**

The online version contains supplementary material available at 10.1186/s12944-022-01711-0.

## Background

Globally, obesity is an increasing health concern. Its morbidity has increased every year[1]. Obesity causes many complications, including cardiovascular disease, stroke, cirrhosis and diabetes[2]. Therefore, new preventative treatment strategies are essential to reduce obesity incidence. The causes of obesity include a variety of causes, including environmental and genetic factors. However, individuals seem to show different levels of susceptibility to obesity[3]. Research has shown that high-fat diets contribute to obesity or obesity resistance in C57BL/6 model mice[4]. It is important to understand different levels of susceptibility to obesity by studying C57BL/6 mice, which have a consistent genetic background. Researchers have examined various factors involved in obesity or obesity resistance. There are several points to explain this. (1) Hormone levels are not the same. Leptin-deficient mice present with lower body weight and higher insulin sensitivity than obese mice[5]. (2) The gut microbiota composition is inconsistent among individuals. The gut microbiota regulates the metabolism of bile acids. Bile acids are closely related to lipid metabolism[6]. (3) The metabolic capacity of the liver among individuals is also inconsistent. For example, the levels of long-chain and highly unsaturated phosphatidylcholine (PC) 40:9 and PC 38:7 are higher in obesity-resistant mouse livers than in obese mouse livers[7]. Triacylglyceride (TG) species with a lower degree of fatty acid chain unsaturation are more highly expressed in obese mouse livers[7]. (4) The amount of activity affects obesity. Physical activity levels are low in overweight and obese individuals, and caloric intake is higher[8]. (5) Different functional states of mitochondria contribute to obesity. A study showed that mitochondrial content and oxidative enzyme activity are lower in obese mice. In contrast, obesity-resistant rats exhibit higher antioxidant molecule and antioxidant enzyme activity levels to maintain cellular redox homeostasis, which favours energy production and efficient energy consumption[9]. (6) Diverse genetic backgrounds are factors. Fabp4-deficient mice showed resistance to obesity and fatty liver[10]. (7) Activity of the nervous system is inconsistent among individuals. Obese rats have higher sympathetic nerve activity[11] and lower brain dopamine levels[12]. However, the cause of obesity resistance remains unclear. More studies are needed. Recently, the majority of the research on obesity resistance has been performed on mice with diverse genetic backgrounds, which may influence the identification of true obesity/obesity resistance factors. There are also other deficiencies in some of these studies. Gut microbiota dysbiosis is commonly considered to be a key factor for obesity. In addition to nutrient digestion and absorption, the gut microbiota produces short-chain fatty acids to regulate intestinal barrier integrity and immune homeostasis[13]. However, the distribution of intestinal microflora varies by intestinal segment. An intestinal microflora study of obesity/obesity resistance is largely based on analyses of fresh faeces or faeces in the caecum. The small intestine is the site of lipid digestion and absorption. The intestinal microflora in fresh faeces or faeces in the caecum cannot fully represent the effect of the whole intestinal flora on obesity/obesity resistance. Recently, there have been many studies on obesity/obesity resistance based on mouse blood and urine metabolomics; despite these efforts, few studies have investigated mechanisms underlying obesity/obesity resistance via small intestine content metabolomics. Studies, such as the research by Wei et al.[12], merely reflect the metabolism in blood, liver and faeces in the caecum, not the small intestine. Although this study revealed a significant relationship between gut microbiota, bile acids and obesity/obesity resistance, the function of other metabolites and related mechanisms in obesity/obesity resistance remain unclear, and further research is needed[12].

In summary, obesity has become a chronic disease and threat to the health of people worldwide. Determining methods for preventive therapy of obesity has become a hotspot for obesity research. Although several studies have explained the mechanism of obesity/obesity resistance, studies on the small intestine microbiota and metabolites of obesity/obesity resistance are rare. Hence, this study arms to investigate the different potential novel biomarkers of obesity/obesity resistance in the small intestine.

## Materials and methods

### Animals and experimental design

All experiments were performed following protocols approved by the Animal Ethics Committee of the Chengdu University of Traditional Chinese Medicine (No. 2021-05). Ninety-four male C57BL/6 mice, 8 weeks (w) old and weighting 20.55 ± 0.82 g, were purchased from Sibeifu (Beijing) Laboratory Animal Technology Co., Ltd. [certificate No. SCXK(Jing)2019-0008]. The mice were maintained in the Experimental Animal Research Center of Chengdu University of Traditional Chinese Medicine with a condition of controlled temperature (22 ± 2℃) and light/dark cycle (12/12 h). Under these conditions, food and water were freely available to all mice. After 1 w of adaptive feeding, six mice were fed a low-fat control diet (caloric composition: 10% fat, 70% carbohydrates, and 20% protein, 3.85 kcal/g total energy content; Art. No. D 12450J from Research Diets, Inc., New Brunswick, NJ, USA), and eighty-eight mice were fed a high-fat diet (HFD) (caloric composition: 60% fat, 20% carbohydrates, and 20% protein, 5.24 kcal/g total energy content; Art. No. D12492 from Research Diets, Inc., New Brunswick, NJ, USA). On the basis of most previous studies, an HFD feeding time of 8 w was chosen to induce obesity. Then, fifty-six obese mice were obtained after HFD feeding and were found to weigh 20% more than those in the low-fat control diet group (LFD group). Among the fifty-six obese mice, six mice were randomly assigned to the HFD-induced obesity prone group (HFDOP). In addition, thirty-two obese resistant mice were obtained, and their body weight did not weigh 20%  more than those in the LFD group. Among the thirty-two obese resistant mice, twelve mice were randomly assigned to the HFD-induced obesity resistance group (HFDOR). All the remaining mice were used for other types of experiments. Following completion of the behavioural assay and blood glucose testing, all the mice were housed single-caged for another 4 w. At the age of 20 w, all the mice were sacrificed.

### Primary obesity index detection

The weights of the mice were recorded weekly using an electronic scale that ranged from 0 to 200 g and was accurate to 0.01 g. Body lengths were measured weekly with callipers. Then, the following formula was used to calculate Lee’s index: Lee’s index = [weight(g) × 1,000] (1/3)/length (cm). The residual food intake per cage was weighed weekly to calculate the average food consumption. Fasting plasma glucose testing was measured at the end of 16 w. After fasting for 12 h, the plasma glucose levels were measured using an Accu-Chek glucose meter (Roche Co., Ltd., Shanghai, China).

### Behavioural tests

Depressive- and anxiety-like behaviours were performed using an open field test. The mice were brought to the testing environment for adaptation for two hours. The open field apparatus consisted of a box measuring 50 × 50 × 40 cm with a white flat bottom plate. After 10 min in the open field apparatus, the mice were removed. The test was automatically video recorded. Then, the data were automatically recorded and analysed with SuperMaze software (XR-Xmaze, Shanghai, China). The entries and total distance travelled in the central area were used for statistical analysis.

An elevated plus maze test was performed to assess depressive- and anxiety-like behaviours. Approximately 60 cm above the ground, two open arms (35 × 5 cm) and two closed arms (35 × 5 × 15 cm) composed the maze apparatus. The mice were positioned in the middle square area and observed for 5 min[14]. Video recordings were automatically taken during the test. Then, the data were automatically recorded and analysed with SuperMaze software (XR-Xmaze, Shanghai, China). The entries and total distance travelled in the open arms were used for statistical analysis.

### Collection of tissues and serum samples

Isoflurane (RWD Life Science Co., Shenzhen, China) was used to deeply anaesthetize the mice, and 500 µl of serum samples were collected by retro-orbital blood sampling. The levels of high-density lipoprotein (HDL), low-density lipoprotein (LDL), total cholesterol (TC), aspartate aminotransferase (AST), TG, and alanine aminotransferase (ALT) were tested with an automatic biochemical analyser (BS-240VET, Mindray Co., Shenzhen, China). The assay kits used in these analyses were purchased from the Chinese Mindray Company (Shenzhen, China). After flash freezing in liquid nitrogen, the jejunum and small intestinal contents were stored at -80 °C for use in further experiments. Epididymal, subcutaneous, perirenal, and mesenteric fat was collected and weighed for analysis.

### Full-length transcriptome sequencing and quantitative polymerase chain reaction (qPCR)

Total RNA was obtained from jejunum tissues using TRIzol (Invitrogen, Carlsbad, CA, USA). In reverse transcription, total RNA (1 µg) was used. According to the protocol of Oxford Nanopore Technologies, cDNA libraries were constructed and sequenced on the PromethION platform (Biomarker Technology Company, Beijing, China). Some genes were verified by qPCR using SYBR qPCR Master Mix (Vazyme Biotech Co.,Ltd., Nanjing, China). Table 1 contains gene-specific primers that were designed and synthesized by FOREGENE Biotech Co., Ltd. (Chengdu, China). Normalization was performed by comparing the expression levels of target genes to β-actin. The 2^−∆∆^CT method was used to determine relative fold changes in mRNA expression.

**Table 1:** Primer sequences

Gene	Forward primer	Reverse primer
Tgfb2	5’-TTGTTACAACACCCTCTGGCT-3’	5’-AGCGGACGATTCTGAAGTAGG-3’
Cxcl10	5’-CCAAGTGCTGCCGTCATTTT-3’	5’-AGCTTCCCTATGGCCCTCAT-3’
Pck1	5’-TGGAAGGTCGAATGTGTGGG-3’	5’-CAGTAAACACCCCCATCGCT-3’
Epha7	5’-GTGGAGTCATGAAGGAGCGA-3’	5’-GACGTACACTGTTCCCGGTT-3’
Spp1	5’-GGAGGAAACCAGCCAAGGACT-3’	5’-AGAATCAGTCACTTTCACCGGG-3’

### Differentially expressed gene (DEG) analysis

The specific analysis method performed for full-length sequencing of the transcriptome is described in the supplementary material. DEGs were analysed, and a heatmap of the DEGs was generated using BMKCloud (www.biocloud.net). A volcano map was generated using OECloud tools available at https://cloud.oebiotech.cn. The DEGs were analysed by https://metascape.org/gp/index.html#/main/step1 for Kyoto Encyclopedia of Genes and Genomes (KEGG) pathway enrichment, and a Sankey diagram of the KEGG pathways was plotted using tools at https://www.bioinformatics.com.cn.

### Gut microbiota sequencing and bioinformatics analysis

An extraction of DNA was performed from 9 small intestinal content samples. Sequencing libraries were prepared by amplifying the 16S rRNA gene with universal primers (27F 5’- AGRGTTTGATYNTGGCTCAG-3’ and 1492R 5’-TASGGHTACCTTGTTASGACTT-3’). After library quality examination, the library was sequenced on a PacBio Sequel platform. The unweighted pair-group method with arithmetic mean analysis (class level) and bar graphs showing genus distribution were performed and graphed using BMKCloud (www.biocloud.net).

### Metabolite extraction and analysis

The intestinal contents (50 mg) were mixed in methanol (1000 µl) with 2-chloro-L-phenylalanine (2 ug/ml final concentration), vortexed for 30 s, sonicated in a 4 °C water bath for 10 min and allowed to stand at -20 °C for 1 h before being centrifuged (15 min, 4 °C, 13,000 rpm). Then, 200 µl of supernatant was used for ultrahigh-performance liquid chromatographic system (UHPLC/QTOF-MS) analysis. The specific LC–MS/MS analysis methods are described in the supplementary material. Partial least-squares–discriminant analysis (PLS–DA) was performed, and graphs were prepared using BMKCloud tools (www.biocloud.net). An analysis of differential metabolite abundance was performed with BMKCloud. Volcano plots, heatmaps based on clustering, and Spearman correlation results were generated using the OECloud tools at https://cloud.oebiotech.cn.

### Statistical analysis

To conduct the statistical analyses and generate data graphs, GraphPad Prism (V9.1.0) software was used. Data with a normally distributed distribution were expressed as mean ± SD. The nonnormal distribution data were shown as median (interquartile distribution). The t-test or one-way ANOVA test was used to quantify the differences between the different groups. Spearman correlation analysis was performed. A *P* value < 0.05 was considered statistically significant for all tests.

## Results

### Primary indices in obesity/obesity resistance

**Fig. 1:**
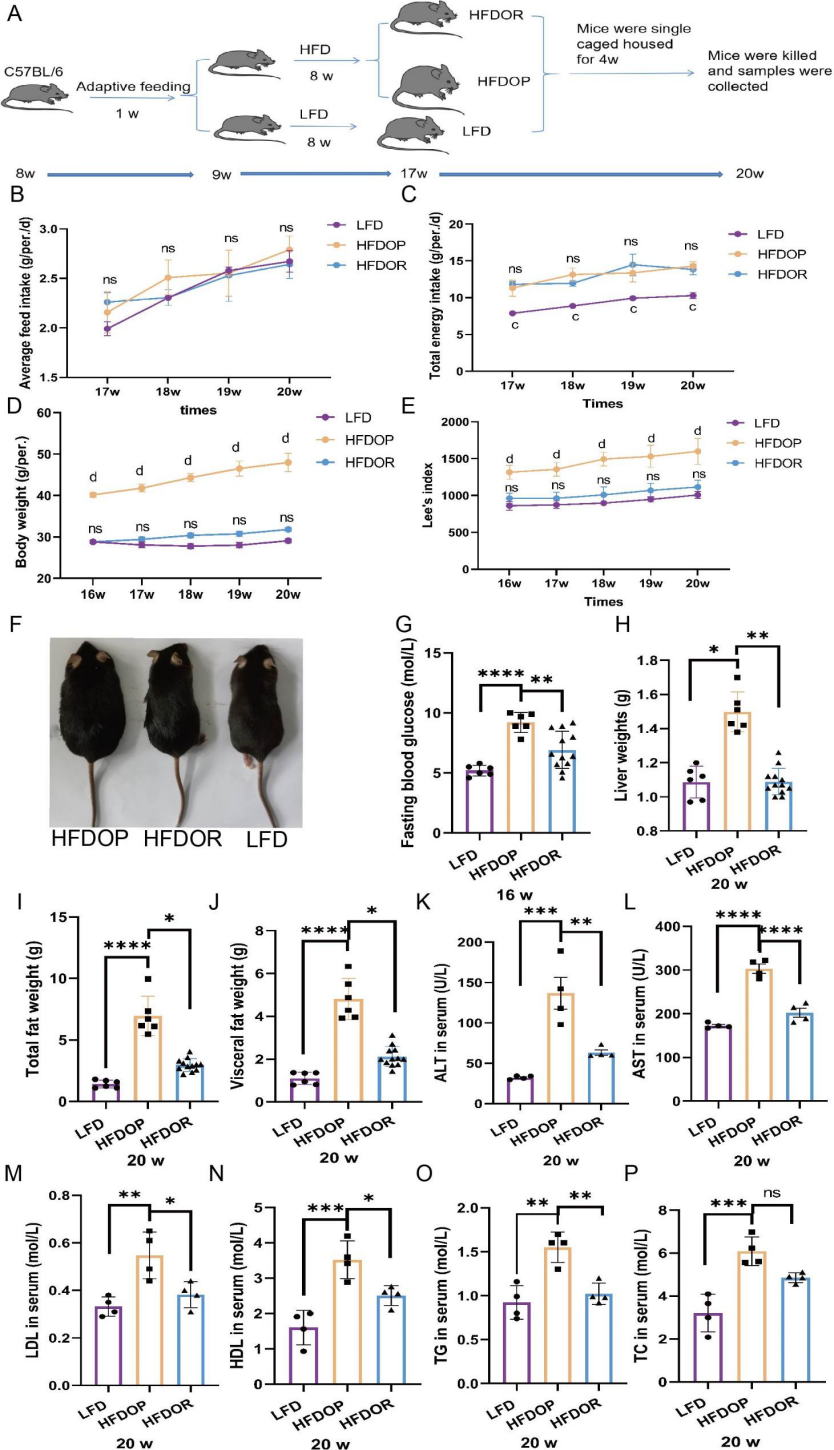
Primary indexes in obesity/obesity resistance mice. **A** Experimental timeline; **B** Average daily feed intake (ns > 0.05, HFDOR compared with HFDOP group); **C** Total energy intake (ns > 0.05, HFDOR compared with HFDOP group; c, *P* < 0.001, HFDOR compared with LFD group); **D** Body weight (ns > 0.05, HFDOR compared with LFD group; d, *P* < 0.0001, HFDOP compared with HFDOR group); **E** Lee’s index (ns > 0.05, HFDOR compared with LFD group; d, *P* < 0.0001, HFDOP compared with HFDOR group); **F** Picture of mice body size (16 w); **G** Fasting blood glucose; **H** - **J** The weight of liver, total fat (epididymal, subcutaneous, perirenal, and mesenteric fat), total visceral fat (perirenal, mesenteric, and epididymal fat); **K** - **P** The ALT, AST, LDL, HDL, TG and TC in serum (20 w) (n = 4). The values are shown as the mean ± SD. Two-group comparisons used an unpaired t test and three-group comparisons used a one-way ANOVA test. (*P < 0.05, **P < 0.01, ***P < 0.001, ****P < 0.0001) (**B** - **J**, n = 6 in the HFDOP and LFD groups, n = 12 in the HFDOR group)

Figure 1A describes the experimental timeline. After being fed a HFD for 8 w, groups of mice were housed in single cages for another 4 w. The body weight and Lee’s index increased continuously in the HFDOP group but not in the HFDOR group. However, the average daily feed and energy intake were no significant differences between the HFDOP and HFDOR mice (both *P* > 0.05) (Fig. 1B, C). After being fed a HFD for 12 w, the mice between the HFDOP and HFDOR groups showed a significant difference in body weight and Lee’s index (both *P* < 0.0001) (Fig. 1D, E). A picture of the mice is shown in Fig. 1 F to show the body size. To exclude the influence of blood glucose levels, fasting blood glucose was tested at the end of the 16-week period. The HFDOP group showed higher fasting blood glucose levels than the HFDOR and LFD groups (Fig. 1G) (*P <* 0.0001 and 0.01, respectively). The weight of the liver, total fat (epididymal, subcutaneous, perirenal and mesenteric fat), and total visceral fat (perirenal, mesenteric and epididymal fat) showed a significant increase in the HFDOP group than the LFD group (Fig. 1 H - J) (*P* < 0.05, 0.0001 and 0.0001, respectively). However, the weights of the liver, total fat and total visceral fat showed a significant decrease in the HFDOR group than the HFDOP group (Fig. 1 H - J) (*P* < 0.01, 0.05 and 0.05, respectively). The HFDOP group showed a significant increase in AST, ALT, HDL, LDL TG and TC levels than the LFD group (Fig. 1 K - P) (*P* < 0.001, 0.0001, 0.01, 0.001, 0.01 and 0.001, respectively). However, the AST, ALT, HDL, LDL and TG levels showed a significant decrease in the HFDOR group than HFDOP group (Fig. 1 K - O) (*P* < 0.01, 0.0001, 0.05, 0.05 and 0.01, respectively). Although the HFDOR group exhibited a lower TC level than the HFDOP group, the difference did not reach statistical significance (Fig. 1P) (*P* > 0.05).

### Obesity resistance is not associated with depression

**Fig. 2:**
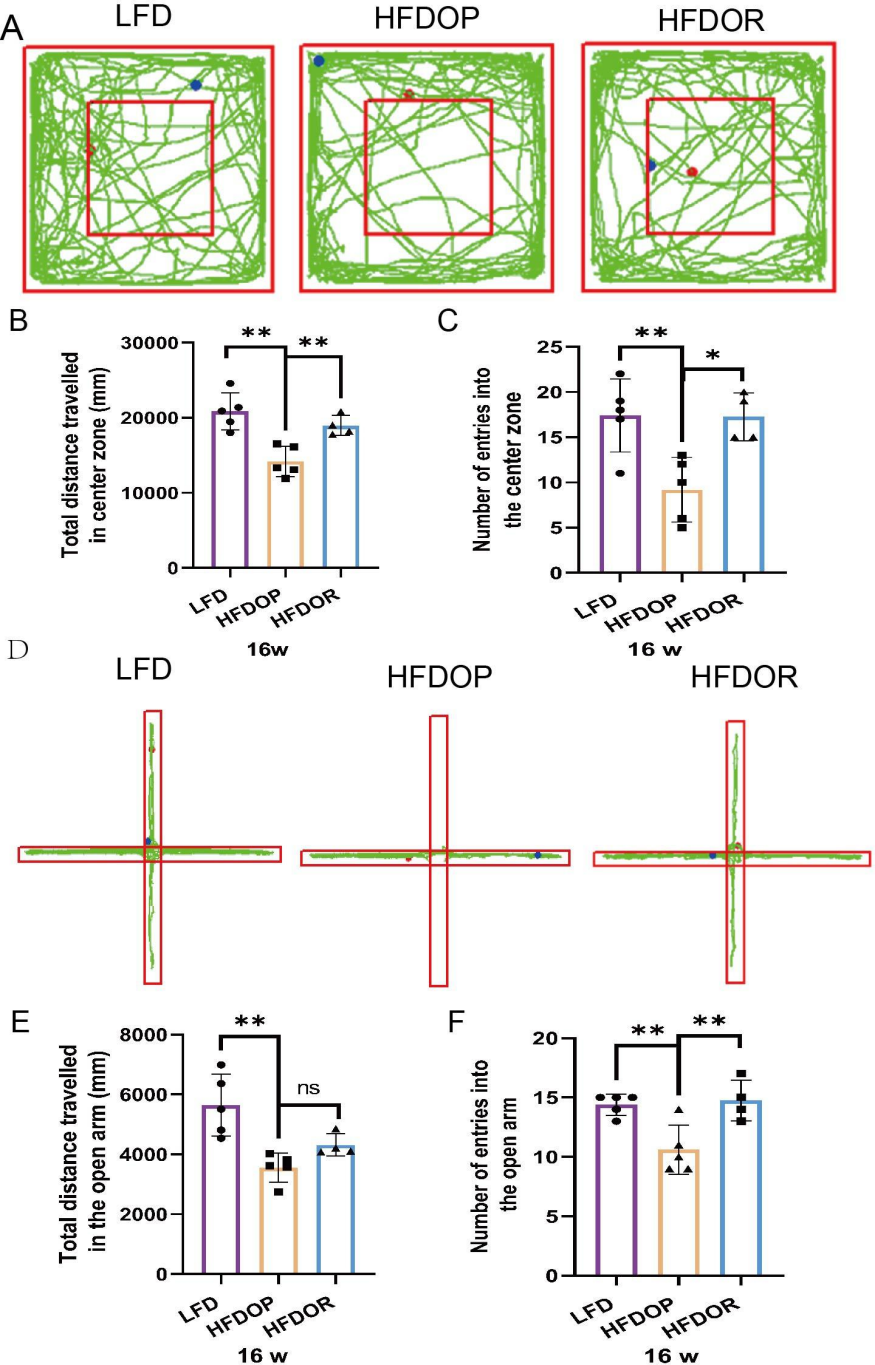
Effects of obesity/obesity resistance on depressive- and anxiety-like behaviors. **A** Open field test; **B**, **C** Statistical analysis of the total distance and entries traveled in the central area; **D** Elevated plus maze test; **E, F** Statistical analysis of the total distance and entries traveled in the open arms. The values are shown as the mean ± SD. Data were analyzed by one-way ANOVA test (*P < 0.05, **P < 0.01, ns > 0.05) (n = 6, outliers were removed)

To rule out the effect of depressive- and anxiety-like behaviours on obesity resistance, an open field test and elevated plus maze test were performed at the end of 16 w. The results of the open field test are shown in Fig. 2 A - C. The HFDOP group travelled considerably less into the central area and entered this area significantly fewer times than the LFD group (Fig. 2B, C) (both *P* < 0.01). The HFDOR group travelled considerably farther into the central area and entered this area more frequently than HFDOP group (Fig. 2B, C) (*P* < 0.01 and *P* < 0.05, respectively). The elevated plus maze test results are shown in Fig. 2D - F. The HFDOP group travelled considerably less into the open arms and entered the open arms less frequently than the LFD group (Fig. 2E, F) (both *P* < 0.01). The HFDOR group travelled farther into the open arms than the HFDOP group, but no statistical significance was observed between the groups (Fig. 2E) (*P* > 0.05). The HFDOR group entered the open arms significantly more frequently than the HFDOP group (Fig. 2 F) (*P* < 0.01).

### Obesity/obesity resistance was associated with the small intestinal microbiota and metabolites

**Fig. 3:**
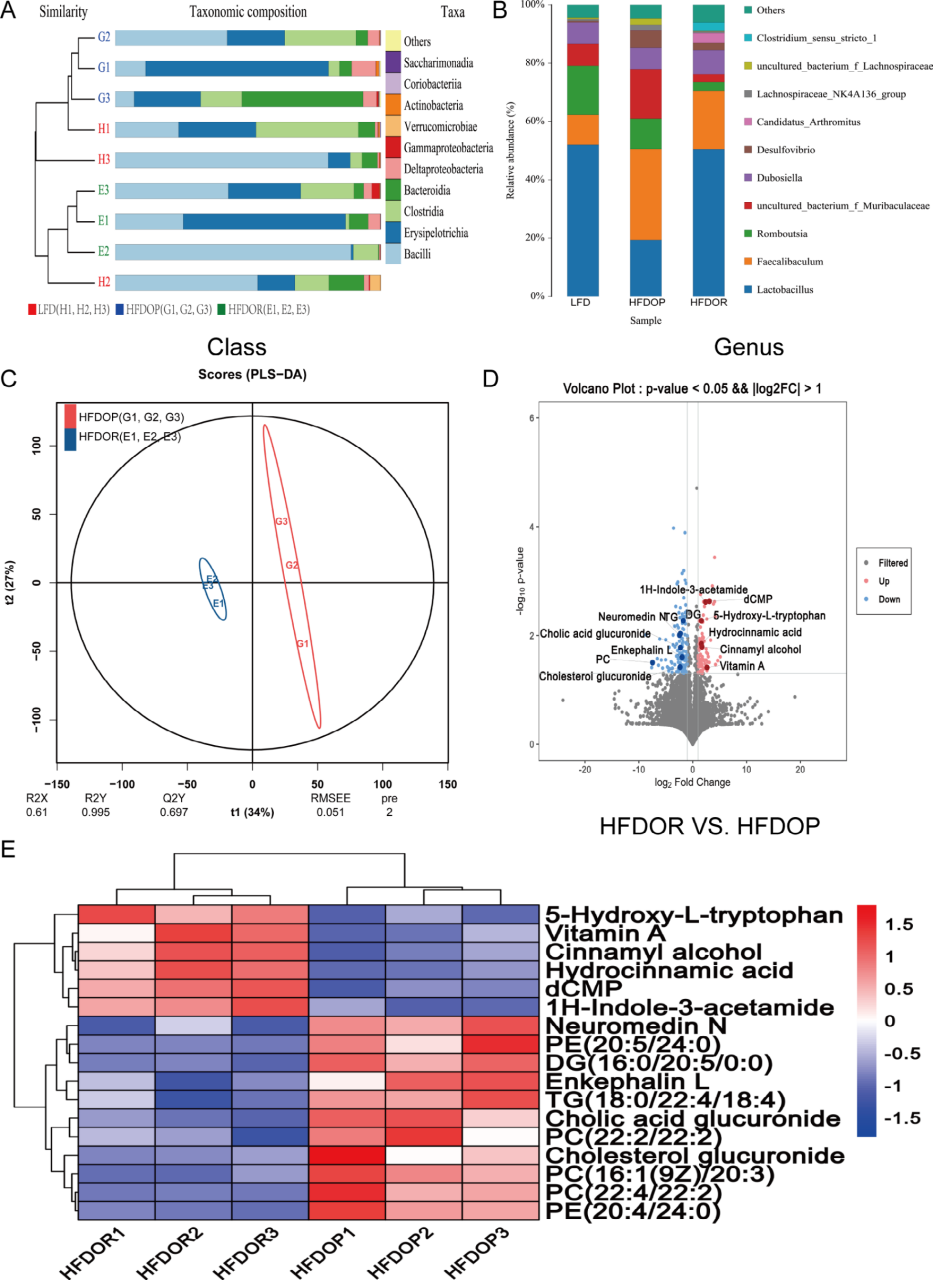
Differential biomarkers of obesity/obesity resistance in the small intestinal microbiota and metabolites. **A** Unweighted pair-group method with arithmetic mean (UPGMA) analysis (class); **B** Bar graph of species distribution (genus); **C** The partial least-squares discriminant analysis (PLS-DA) of small intestinal metabolites; **D** Volcano plots of differentially abundant metabolites. **E** Heatmap of the relative contents of differential metabolites of interest. Red indicates a higher amount of a metabolite and blue indicates a lower amount of a metabolite. (n = 3)

**Fig. 4:**
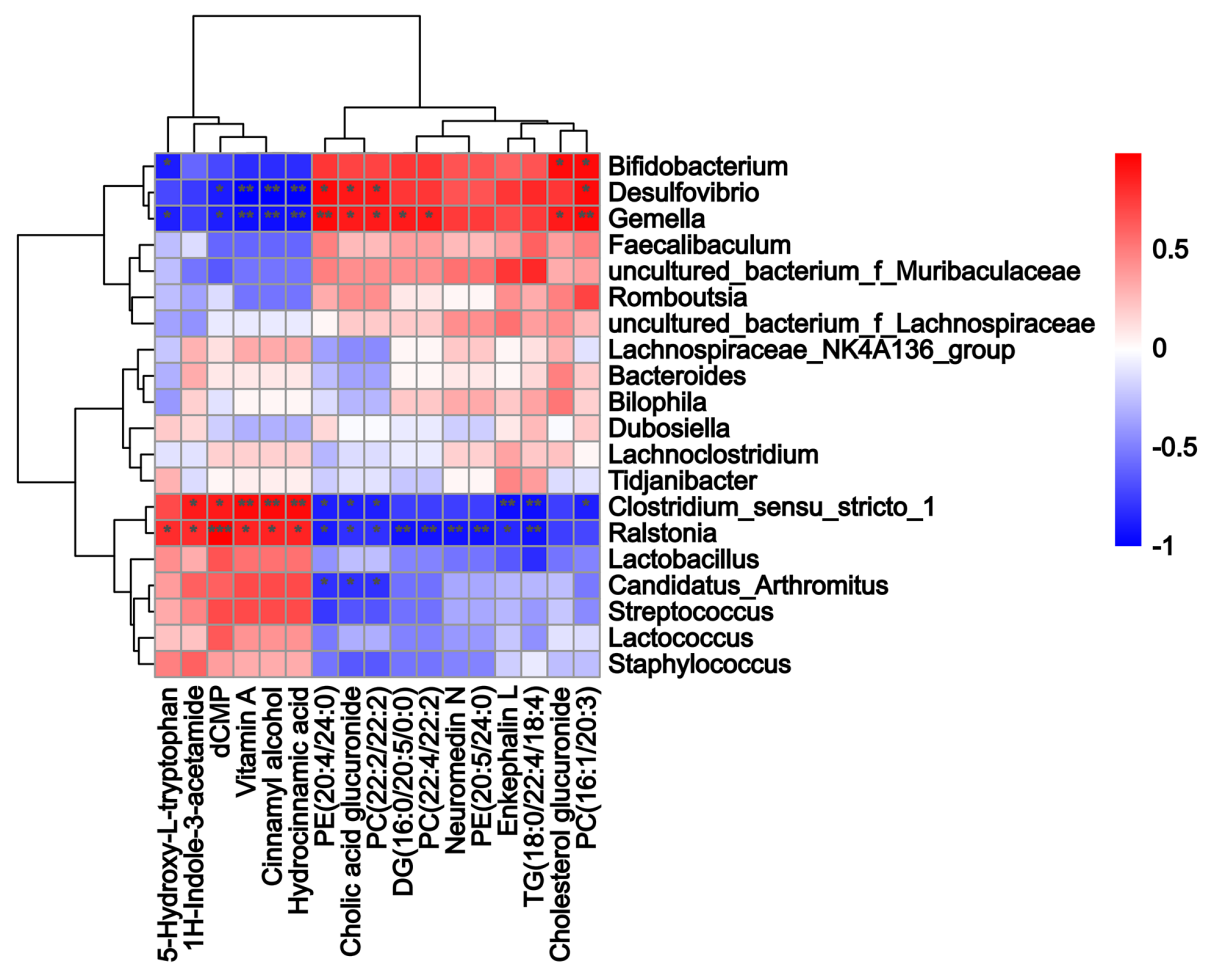
Spearman correlation analysis between the microbiota and metabolites in the small intestine. Red indicates an increase, and blue indicates a decrease in that parameter. (*P < 0.05, **P < 0.01, ***P < 0.001)

Many studies have confirmed that the intestinal microbiota is associated with obesity/obesity resistance, but most of these prior studies were based on fresh faeces or faeces in the caecum not the small intestine. In this study, we measured small intestinal contents using 16 S genome sequencing and an untargeted metabolomics approach. According to the clustering analysis at the class level, the HFDOR group small intestinal microbiota was more similar to that of the LFD group than to that of the HFDOP group (Fig. 3 A). Figure 3B; Table 2 show the relative abundance of the top 10 genera identified in each group. The relative abundance of *Muribaculaceae*, *Faecalibaculum*, *Desulfovibrio*, and *Lachnospiraceae* increased in the HFDOP group than that in the LFD and HFDOR groups, but the differences were not statistically significant (Fig. 3B; Table 2). The relative abundance of *Clostridium* and *Lactobacillus* was higher in the LFD and HFDOR groups than HFDOP group, but only the difference in *Clostridium* level was statistically significant (Fig. 3B; Table 2) (*P* < 0.0001). The results of small intestinal content untargeted metabolomics are shown in Fig. 3 C - E, and statistical parameters are shown in Supplementary Fig. 1 A - Q. The PLS–DA revealed a clear separation between the HFDOR and HFDOP groups (Fig. 3 C). There were a total of 314 differential metabolites among the cationic and anionic metabolites (Fig. 3D). The metabolites of interest are marked in a volcano plot (Fig. 3D) (*P* < 0.05, |logFC| > 1.5 and VIP values  >  1). The metabolite relative contents of 5-hydroxy-L-tryptophan (5-HT), vitamin A, cinnamyl alcohol, dCMP, 1 H-indole-3-acetamide, and hydrocinnamic acid metabolites increased significantly in the HFDOR group than that in the HFDOP group (Fig. 3E, Supplementary Fig. 1 A - F) (*P* < 0.01, 0.05, 0.01, 0.01, 0.01, and 0.01, respectively). However, the relative metabolite contents of phosphatidylethanolamine (PE) (20:5/24:0), diacylglycerol (DG) (16:0/20:5/0:0), enkephalin L, TG (18:0/22:4/18:4), neuromedin N, PC (22:2/22:2), cholic acid glucuronide, PC (16:1(9Z)/20:3), PC (22:4/22:2), PE (20:4/24:0) and cholesterol glucuronide were higher in the HFDOP group than in the HFDOR group, but only cholesterol glucuronide had not statistical significance (Fig. 3E, Supplementary Fig. 1G - Q) (*P* < 0.05, 0.001, 0.05, 0.01, 0.01, 0.05, 0.01, 0.01, 0.01, respectively). A small intestinal microbiota and metabolite correlation analysis was performed to determine the Spearman correlation (Fig. 4). Statistically significant correlations were found between the small intestinal microbiota and metabolites. *Desulfovibrio*, *Bifidobacterium*, and *Gemella* all correlated positively with PC, DG and cholic acid glucuronide, while these genera correlated negatively with vitamin A, dCMP, cinnamyl alcohol, 5-HT and cinnamon acid glucuronide (Fig. 4). *Clostridium_*sensu*_stricto_1* and *Ralstonia* correlated positively with vitamin A, dCMP, cinnamyl alcohol, 5-HT, 1 H-indole-3-acetamide and hydrocinnamic acid and correlated negatively with PC, PE, TG, DG, neuromedin N, enkephalin L and cholesterol glucuronide (Fig. 4).

**Table 2:** Top 10 genera detected in small intestine microbiota

genus	LFD group Median (upper-quartile, lower-quartile)	HFDOP group Median (upper-quartile, lower-quartile)	HFDOR group Median (upper-quartile, lower-quartile)
*Lactobacillus*	0.53236(0.801, 0.23504)	0.10645(0.41528, 0.0632)	0.34856(0.88345, 0.25058)
*Faecalibaculum*	0.07334(0.16542,0.05831)	0.19457(0.57535,0.16457)	0.18364(0.44296, 0.00409)
*Romboutsia*	0.07753(0.36666, 0.02674)	0.0368(0.25454, 0.02236)	0.01147(0.07266, 0.0019)
*uncultured_bacterium_f_Muribaculaceae*	0.062(0.12326, 0.05597)	0.04381(0.4205, 0.04255)	0.01533(0.06883, 0.00131)
*Dubosiella*	0.06693(0.12615, 0.02426)	0.05532(0.11376, 0.0523)	0.08902(0.16877, 0.00424)
*Desulfovibrio*	0.005035(0.01038, 0)	0.04358(0.08893,0.04196)	0.02786(0.04523, 0.00029)
*Candidatus_Arthromitus*	0(0, 0)	0(0.00450, 0)	0.00409(0.09344, 0.001)
*Lachnospiraceae_NK4A136_group*	0.00791(0.00861, 0.00774)	0.00219(0.05082, 0)	0.00526(0.01046, 0)
*uncultured_bacterium_f_Lachnospiraceae*	0.00511(0.00633, 0.00133)	0.00872(0.0591,0.00088)	0.00161(0.0028, 0)
*Clostridium_sensu_stricto_1*	0 (0.00015, 0)	0(0, 0)	0.00412675(0.07843, 0.00017)****

Note: The relative abundances of the top 10 genera are shown as the median (upper-quartile, lower-quartile). Compare with HFDOP group, ****P < 0.0001, n = 3

### Mice with obesity/obesity resistance exhibited different transcription levels in the jejunum

**Fig. 5:**
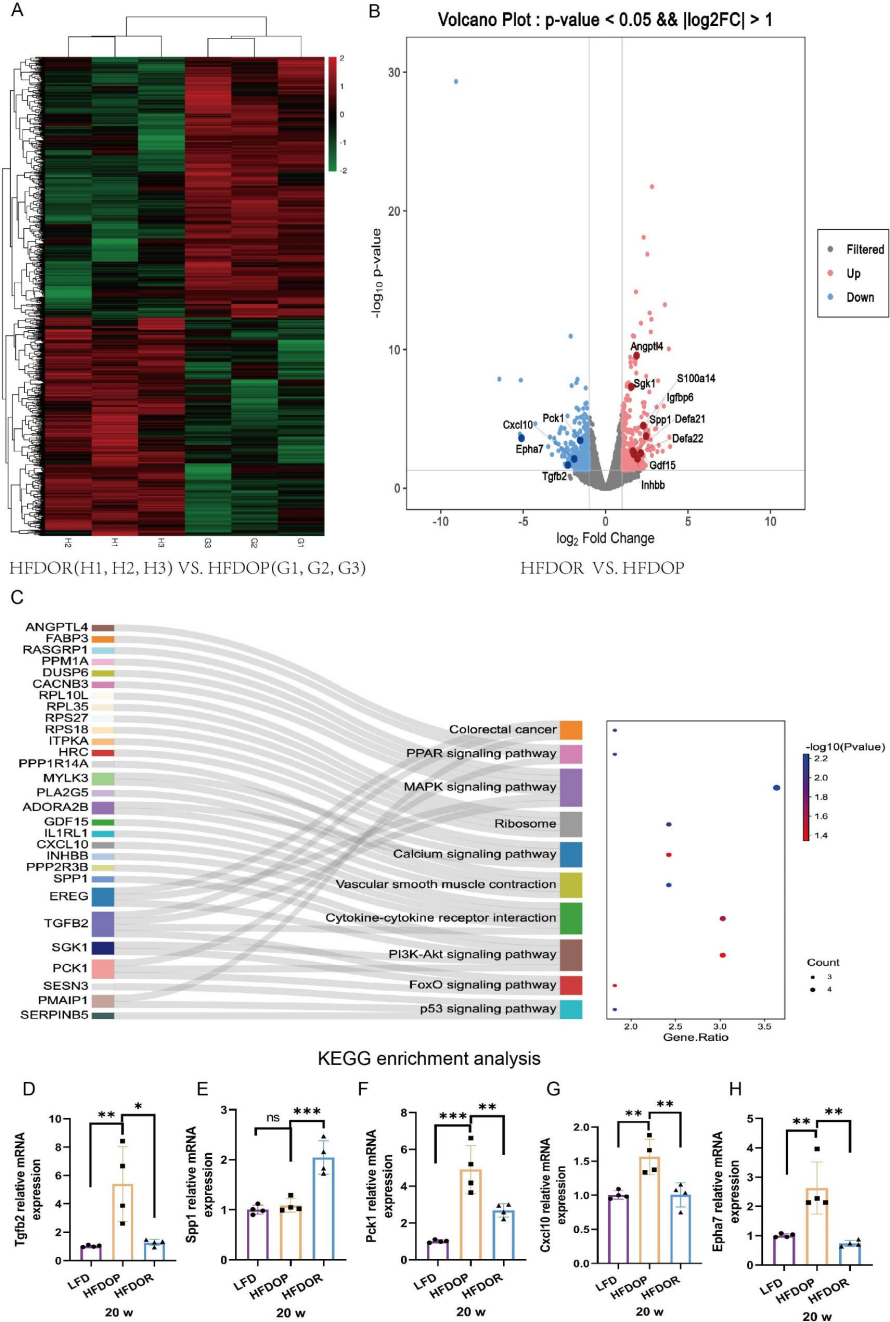
Different transcript levels of obesity/obesity resistance in the jejunum. **A** Heatmap of DEGs in the HFDOP and HFDOR groups; **B** Volcano plot of DEGs in the HFDOP and HFDOR groups; **C** Sankey diagram for KEGG pathways; **D** - **H** Some DEGs were validated by qPCR (n = 4). The values are shown as the mean ± SD. Data were analyzed by one-way ANOVA test. (ns > 0.05, *P < 0.05, **P < 0.01, ***P < 0.001)

To further explore the reason for obesity/obesity resistance, gene transcript levels in the jejunum tissues were determined using a full-length transcriptomic approach. A total of 1645 DEGs (750 upregulated and 895 downregulated) were identified, and among these DEGs, 331 showed significantly different expression (*P* < 0.05, |logFC| > 1.5) (167 upregulated and 164 downregulated) (Fig. 5A, B). Some genes of interest are marked in the volcano plot (Fig. 5B). Among the 331 significant DEGs, 30 genes were enriched in 11 KEGG pathways (Min Overlap = 3, a *P* value cut-off = 0.05, min enrichment = 1.5). The enriched genes and 10 most enriched KEGG pathways are shown in Fig. 5 C. These genes were significantly enriched in mitogen-activated protein kinase (MAPK), cytokine and phosphatidylinositol 3’ kinase/protein kinase B (PI3K/Akt) signalling pathways (Fig. 5 C). The DEGs of interest were individually validated by qPCR (Fig. 5D - H). According to the results, Tgfb2, Spp1, Pck1, Cxcl10 and Epha7 showed a consistent mRNA expression pattern, as determined on the basis of the full-length transcriptome data (Fig. 5B, D - H).

### Correlation analysis of significant DEGs and metabolite or microbiota contents

**Fig. 6:**
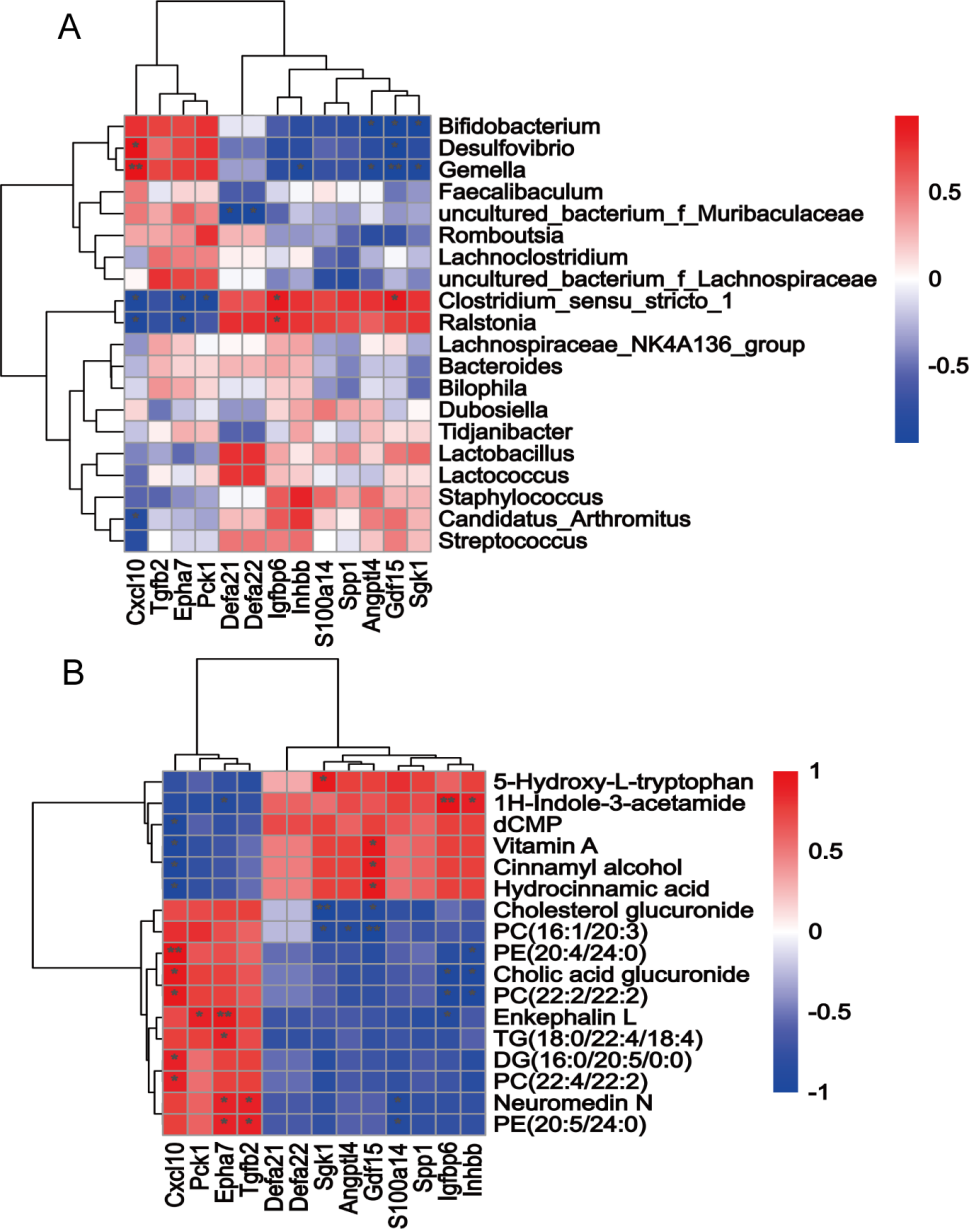
** A** Spearman correlation analysis between genes of interest and the microbiota in the small intestinal; **B** Spearman correlation analysis between genes of interest and metabolites in the small intestinal. Red indicates an increase and blue indicates a decrease in that parameter. (*P < 0.05, **P < 0.01)

The Spearman correlation analysis of DEGs and microbiota can be found in Fig. 6 A. *Desulfovibrio*, *Bifidobacterium*, and *Gemella* correlated positively with Cxcl10 and negatively with Sgk1, Angptl4, and Gdf15 levels. *Clostridium_sensu_stricto_1* and *Ralstonia* correlated positively with Igfbp6 and Gdf15 and negatively with Epha7, Pck1 and Cxcl10 (Fig. 6 A). In addition, Defa21 and Defa22 showed a negative correlation with *uncultured_bacterium_f_Muribaculaceae* (Fig. 6 A). The correlation analysis results for DEGs and metabolites can be found in Fig. 6B. 1 H-indole-3-acetamide has a significant positive association with Igfbp6 and Inhbb levels and a negative association with Epha7. The metabolite 5-HT showed a significant positive correlation with Sgk1. Vitamin A, cinnamyl alcohol and hydrocinnamic acid showed a significant positive association with Gdf15 and a negative association with Cxcl10. The metabolites PC, PE, TG, DG, enkephalin L, neuromedin N, cholic acid glucuronide and cholesterol glucuronide showed a significant positive association with Cxcl10, Pck1, Epha7 and Tgfb2 and a negative association with Inhbb, Igfbp6, S100a14, Gdf15, Angptl4 and Sgk1 (Fig. 6B).

## Discussion

Excessive food intake combined with a high calorie and sedentary lifestyle greatly increases the risk for developing obesity. Obesity shows a significant positive correlation with the morbidity of liver cirrhosis, stroke, type 2 diabetes and coronary heart disease[15, 16], which seriously threatens human health. Therefore, novel obesity preventive and therapeutic modalities need to be urgently explored. A study showed that among individuals with the same lifestyle, living in the environment, and meeting the same dietary conditions, susceptibility to obesity differed[17]. Even overweight/obese individuals can maintain a metabolically healthy phenotype and show no increased risk of metabolic complications[17]. Hence, exploring the reason that individuals under the same conditions develop obesity/obesity resistance is a research hotspot in obesity prevention and treatment.

A HFD did not induce higher blood lipids in obesity resistance. After being fed a HFD, obesity and obesity resistance models were established. Lower levels of TG, TC, HDL, LDL, blood glucose, AST and ALT were observed in the obesity resistance model than in the obesity model, which are similar to other studies[4, 18]. A HFD caused depression and anxiety-like behaviours in obese mice [19, 20], but similar behaviours were not observed in obesity-resistant mice in this study. This result was associated with low levels of serum TC, LDL, TG and blood glucose in the obesity-resistant mice[21–23]. A previous study explicitly stated that high TG levels exerted a causal effect on depression[21]. TG has been reported to be negatively correlated with the thickness of the anterior cingulate cortex[22], which is associated with emotion regulation[23]. High LDL, HDL and blood glucose levels are risk factors for HFD-induced brain impairment[24]. An increased lipid load and abnormal neuronal morphology were observed in the frontal cortex and hippocampus[19, 20]. Under this condition, microglia are overactivated via the Toll-like receptor 4/nuclear factor-κB signalling pathway, and the levels of interleukin-1β, interleukin-6 and tumour necrosis factor-α significantly increase, inducing depressive and anxiety-like behaviours[19, 20]. Hence, the HFDOR group showed lower inflammation and brain impairment and fewer depressive and anxiety-like behaviours. A study showed that serum TC, HDL and LDL levels were positively correlated with *Faecalibaculum*[25]. The TC, HDL, LDL and *Faecalibaculum* levels increased significantly in the HFDOP group than that in the HFDOR group. A greater Faecalibaculum abundance in the HFDOP group was associated with higher TC, HDL and LDL levels. However, the detailed mechanisms of these associations require further investigation.

The small intestine microbiota is closely associated with obesity resistance. *Lactobacillus* can improve body weight and insulin resistance, increase fatty acid oxidation and decrease lipogenesis in obesity through the sterol regulatory element binding protein-1 and peroxisome proliferator-activated receptor α signalling pathways[26]. In this study, the *Lactobacillus* and *Clostridium* contents were clearly increased in the small intestine of the HFDOR group, which are reasons for obesity resistance[27, 28]. However, the *Faecalibaculum*, *Lachnospiraceae*, *Muribaculaceae* and *Desulfovibrio* contents were distinctly increased in the small intestine of the HFDOP group, similar to previous studies[29–31]. *Lachnospiraceae*, the dominant bacterial group in the HFDOP group, was associated with an increased relative abundance of other genera related to HFD-induced obesity[31]. *Desulfovibrio* is a dominant bacterial group in obesity[30], and the present study corroborated this finding. *Desulfovibrio* is a sulfate-reducing bacterium that can produce lipopolysaccharides and induce inflammatory and intestinal mucosa damage[32, 33]. *Desulfovibrio* showed a positive association with serum levels of lipopolysaccharide[33], which causes low-grade chronic systemic inflammation in obesity[34]. Obesity and type 2 diabetes induce impaired immune function and reduced secretion of IgA[35]. The reduced secretion of IgA led to reduced *Clostridium* colonization and gut microbial dysbiosis[35]. In addition, in a previous study, culture supernatants of *Desulfovibrio* showed upregulated expression of CD36, and the culture supernatants of *Clostridium* showed downregulated expression of CD36[35]. CD36 is a key factor promoting lipid absorption in the small intestine[35]. The greater the expression of CD36 is, the greater the lipid absorption in the small intestine.

Small intestine metabolites are closely associated with obesity resistance. Gut microbes can produce some monoamine neurotransmitters, such as γ-aminobutyric acid[36], tryptamine[37], catecholamine and 5-HT[38], and promote intestinal enteroendocrine cell secretion. All outcomes can affect the enteric nervous system either directly or indirectly, and the local signals can be relayed to brain regions to orchestrate feeding behaviours and modulate appetite and satiety[39]. Metabolites connect the microbiota and the host. Fat digestion products such as PC, TG, and PE were higher in the HFDOP group and were significantly correlated with *Desulfovibrio* and *Gemella* contents in this study. Fatty acid production in the small intestine has been previously associated with *Desulfovibrio*[40]. In addition, the metabolites neuromedin N and enkephalin L were negatively associated with *Clostridium* in this study. Neuromedin N can act on intestinal mucosa, stimulate gastric acid and insulin secretion, inhibit gastric emptying and small intestinal motility and promote fatty acid absorption in the jejunum[41, 42]. Neuromedin N also regulates intestinal sympathetic nervous activity, and its signal is relayed to brain regions, inhibiting gut motility[43]. These results showed that the microbiota-gut-brain axis is involved in obesity resistance formation processes. Enkephalin L, an endogenous opioid distributed in the enteric nervous system (ENS) and enterochromaffin cells, is associated with the intestinal immune response and can inhibit intestinal motility and secretion[44, 45]. Fu et al. [46] proved that a HFD slows intestinal motility. A decrease in intestinal motility increases the time that lipids remain in the intestine and leads to increased lipid uptake. The metabolite 5-HT showed a significant increase in the HFDOR group in this study. The gastrointestinal tract is the prime site of 5-HT synthesis, and a crucial role of the gut microbiota is regulation of 5-HT synthesis [47, 48]. 5-HT is a nerve growth factor that performs functions during ENS development[49]. 5-HT also reduces intestinal mucosal inflammation[50], increases intestinal motility[51], and reduces the stay of lipids in the intestine and absorption.

The metabolite of 1 H-indole-3-acetamide is an indole. A previous study showed that the bacterial metabolite indole stimulated glucagon-like peptide secretion by L-cells[52]. A metabolite indole from *Lactobacillus* regulates interleukin-22 release by activating the aryl hydrocarbon receptor signalling pathway and protects small intestinal mucosal homeostasis[53]. An interesting metabolite, cinnamyl alcohol, was found in the intestine in the HFDOR group. Long-term HFD feeding causes adipocyte hyperplasia[54]; however, cinnamyl alcohol inhibits adipocyte hyperplasia[55] and promotes lipid metabolism via the PPAR signalling pathway[56]. Hence, cinnamyl alcohol is considered a promising candidate for preventing and treating obesity. The cinnamyl alcohol content was significantly and positively associated with the relative abundance of *Clostridium_sensu_stricto_1* in this study. The increased abundance of *Clostridium_sensu_stricto_1* in the HFDOR group may benefit cinnamyl alcohol production and loss of body weight.

The microbiota and metabolites in the small intestine affect intestinal transcription. DEGs were significantly enriched in MAPK, cytokine and PI3K/Akt signalling pathways. The gene Cxcl10 was enriched in these signalling pathways and was significantly and positively correlated with *Desulfovibrio* and lipid levels. A study showed that Cxcl10 was produced by microglia and induced neuronal cell damage[57]. In other words, Cxcl10 may be a potential target of *Desulfovibrio*-induced and lipid-induced damage in the small intestine. *Desulfovibrio*[58] and lipid[59] are associate with ENS damage and slowing down gut motility. This study showed that this may be related to upregulated Cxcl10 expression in the small intestine. Gdf15 showed a significant positive association with vitamin A and *Clostridium_sensu_stricto_1*. Gdf15 modulates inflammation by regulating TG metabolism[60]. Igfbp6 also showed a significant positive association with *Clostridium_sensu_stricto_1* and 1 H-indole-3-acetamide. A previous study showed that Igfbp6 was expressed at lower levels in obesity and was positively correlated with leptin, glucagon-like peptide 1 and cholecystokinin[61]. Spp1 and Sgk1 were higher expression and Pck1 was lower expression in the HFDOR group, and these genes were enriched in the PI3K/Akt signalling pathway. Pck1 is a rate-limiting enzyme in gluconeogenesis, and overexpression of Pck1 has been previously shown to lead to obesity and an increase in triglyceride and esterification of fatty acids[62]. In addition, in this study, S100a14, Defa 22 and Defa21 were more highly expressed in the HFDOR group. Defa 22 and Defa21, which are defensins, show a broad spectrum of antimicrobial activity and inhibit damage to the intestinal mucosa by harmful bacteria[63]. These factors may favour obesity resistance.

The intestinal microbiota, gut metabolites and intestine gene transcription create a web of interactions, causing individual susceptibility to obesity. In contrast to other obesity-resistance relevant studies[12, 64], this study is the first to jointly analyse intestinal microbiota, gut metabolites and intestinal gene transcription. These results fill a gap in knowledge about obesity resistance with respect to small intestine factors. However, these results still need to be verified via further study.

### Study strengths and limitations

There are several strengths in this study. First, of the many studies on obesity resistance, few studies have focused on the microbiota, metabolites and transcription in the small intestine. Second, the results of a multiomics integrative analysis showed that Clostridium, 5-HT, enkephalin L, neuromedin N, cinnamyl alcohol and 1 H-indole-3-acetamide were valuable for the treatment/prevention of obesity, but their effects need to be confirmed in a larger study. In addition, this study has some limitations. The most important limitation was related to the number of mice. Although the number was sufficient for the analyses, the number of mice was inconsistent between groups. Moreover, the results of multiomics analysis need to be verified.

## Conclusion

In summary, intestinal microbiota, gut metabolites and intestine genes interact with each other. The intestinal microbiota *Clostridium*, *Desulfovibrio* and *Lachnospiraceae* may act directly on intestinal mucosa, and their metabolites 5-HT, enkephalin L and neuromedin N may regulate the ENS and relay the signals to the brain. Therefore, the “microbiota-gut-brain” axis may contribute to obesity resistance. In addition, Cxcl10 is a potential target of HFD-induced ENS damage. Future preventive and therapeutic obesity targets might include 5-HT, enkephalin L and neuromedin N, cinnamyl alcohol and 1 H-indole-3-acetamide. Probiotic supplements, especially Clostridium, might be helpful for treating obese patients or for obesity prevention.

## Electronic supplementary material

Below is the link to the electronic supplementary material.

Supplementary Material 1

## Data Availability

All the primary data presented in the study are included in the article/supplementary materials, and further inquiries can be directed to the corresponding author.

## Abbreviations

HFD: High fat diet.
HDL: High density lipoprotein.
LDL: Low density lipoprotein.
TC: Total cholesterol.
TG: Triglyceride.
AST: Aminotransferase.
ALT: Alanine aminotransferase.
ENS: Enteric nervous system.
Cxcl10: CXC motif chemokine 10.
Defa21: defensin, alpha 21.
PI3K/Akt: Phosphatidylinositol 3’ kinase / protein kinase B.
5-HT: 5-hydroxytryptamine.
Spp1: Secreted phosphoprotein1.
Igfbp6: Insulin-like growth factor-binding protein 6.
Gdf15: Growth differentiation factor 15;
Sgk1: Serum/glucocorticoid-regulated kinase 1
Pck1: Phosphoenolpyruvate carboxykinase 1
MAPK: Mitogen-activated protein kinase
DEGs: Differentially expressed genes
PPAR: Peroxisome proliferators-activated receptor
CD36: CD36 antigen
PC: Phosphatidylcholine
PE: Phosphatidylethanolamine
dCMP: Deoxycytidine monophosphate
