# Supplementary material for: Potential novel biomarkers in small intestine for obesity/obesity resistance revealed by multi-omics analysis
Source: Lipids Health Dis. 2022 Oct 8;21:98. doi: 10.1186/s12944-022-01711-0 (PMC9547412; doi:10.1186/s12944-022-01711-0)
Supplement: Supplementary file 1 — Supplementary Material 1 [file 12944_2022_1711_MOESM1_ESM.docx]

**Supplementary materials**

**1 The specific analysis method of full-length sequencing transcriptome**

1.1 Oxford Nanopore Technologies Long Read Processing

Raw reads were first filtered with minimum average read quality score=7 and minimum read length=500bp. Ribosomal RNA were discarded after mapping to rRNA database. Next, full-length, non-chemiric (FLNC) transcripts were determined by searching for primer at both ends of reads. Clusters of FLNC transcripts were obtained after mapping to reference genome with mimimap2, and consensus isoforms were obtained after polishing within each cluster by pinfish.

1.2 Remove redundant

Consensus sequences were mapped to reference genome using minimap2. Mapped reads were further collapsed by cDNA_Cupcake package with min-coverage=85% and min-identity=90%. 5’ difference was not considered when collapsing redundant transcripts.

1.3 Find fusion transcript

The criteria for fusion candidates is that a single transcript must:

(1) must map to 2 or more loci

(2) minimum coverage for each loci is 5% and minimum coverage in bp is >= 1 bp

(3) total coverage is >= 95%

(4) distance between the loci is at least 10kb

**2 Metabolite**

2.1 LC-MS/MS Analysis

LC-MS/MS analyses were performed using an UHPLC system (1290, Agilent Technologies) with a UPLC BEH Amide column (1.7μm 2.1*100mm, Waters) coupled to TripleTOF 5600 (Q-TOF, AB Sciex). The mobile phase consisted of 25mM NH4OAc and 25mM NH4OH in water（pH=9.75）(A) and acetonitrile (B) was carried with elution gradient as follows: 0 min, 95% B; 7min, 65% B; 9 min, 40% B; 9.1 min, 95% B; 12 min, 95% B, which was delivered at 0.5mL min-1. The injection volume was 3μL. The Triple TOF mass spectrometer was used for its ability to acquire MS/MS spectra on an information-dependent basis (IDA) during an LC/MS experiment. In this mode, the acquisition software (Analyst TF 1.7, AB Sciex) continuously evaluates the full scan survey MS data as it collects and triggers the acquisition of MS/MS spectra depending on preselected criteria. In each cycle, 12 precursor ions whose intensity greater than 100 were chosen for fragmentation at collision energy (CE) of 30 V (15 MS/MS events with product ion accumulation time of 50 msec each). ESI source conditions were set as following: Ion source gas 1 as 60 Psi, Ion source gas 2 as 60 Psi, Curtain gas as 35 Psi, source temperature 650℃, Ion Spray Voltage Floating (ISVF) 5000 V or -4000 V in positive or negative modes, respectively.

2.2 Data preprocessing and annotation

MS raw data (.d) files were converted to the mzXML format using ProteoWizard, and processed by R package XCMS (version 3.2). The preprocessing results generated a data matrix that consisted of the retention time (RT), massto-charge ratio (m/z) values, and peak intensity. R package CAMERA was used for peak annotation after XCMS data processing. In-house MS2 database was applied in metabolites identification.

1. **Supplementary Figure 1**

**
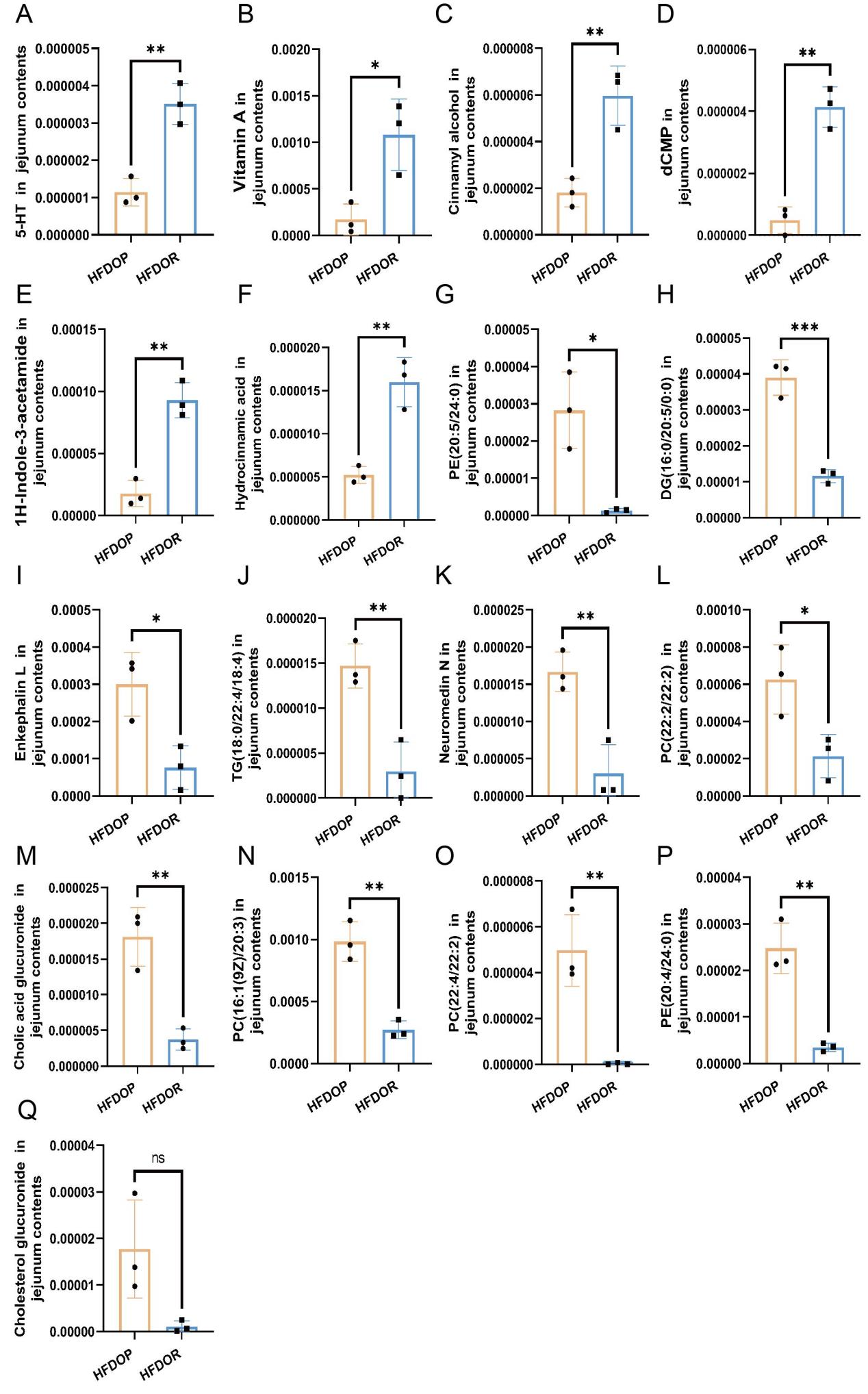
**

**Supplementary Figure 1** Statistical analysis of the relative contents of differential metabolites of interest. The values are shown as the mean ± SD. Data were analyzed by unpaired t test (*P<0.05, **P<0.01, ***P<0.001, ns > 0.05).

| Gene | Forward primer | Reverse primer |
| --- | --- | --- |
| Tgfb2 | 5’-TTGTTACAACACCCTCTGGCT-3’ | 5’-AGCGGACGATTCTGAAGTAGG-3’ |
| Cxcl10 | 5’-CCAAGTGCTGCCGTCATTTT-3’ | 5’-AGCTTCCCTATGGCCCTCAT-3’ |
| Pck1 | 5’-TGGAAGGTCGAATGTGTGGG-3’ | 5’-CAGTAAACACCCCCATCGCT-3’ |
| Epha7 | 5’-GTGGAGTCATGAAGGAGCGA-3’ | 5’-GACGTACACTGTTCCCGGTT-3’ |
| Spp1 | 5’-GGAGGAAACCAGCCAAGGACT-3’ | 5’-AGAATCAGTCACTTTCACCGGG-3’ |

**4 Supplementary Tables**

**Supplementary Table 1. Primer sequences.**

**Supplementary Table 2. Top 10 genera detected in intestine microbiota**

| genus | LFD group  Median (upper-quartile, lower-quartile) | HFDOP group  Median (upper-quartile, lower-quartile) | HFDOR group  Median (upper-quartile, lower-quartile) |
| --- | --- | --- | --- |
| *Lactobacillus* | 0.53236(0.801, 0.23504) | 0.10645(0.41528, 0.0632) | 0.34856(0.88345, 0.25058) |
| *Faecalibaculum* | 0.07334(0.16542,0.05831) | 0.19457(0.57535,0.16457) | 0.18364(0.44296, 0.00409) |
| *Romboutsia* | 0.07753(0.36666, 0.02674) | 0.0368(0.25454, 0.02236) | 0.01147(0.07266, 0.0019) |
| *uncultured_bacterium_f_Muribaculaceae* | 0.062(0.12326, 0.05597) | 0.04381(0.4205, 0.04255) | 0.01533(0.06883, 0.00131) |
| *Dubosiella* | 0.06693(0.12615, 0.02426) | 0.05532(0.11376, 0.0523) | 0.08902(0.16877, 0.00424) |
| *Desulfovibrio* | 0.005035(0.01038, 0) | 0.04358(0.08893,0.04196) | 0.02786(0.04523, 0.00029) |
| *Candidatus_Arthromitus* | 0(0, 0) | 0(0.00450, 0) | 0.00409(0.09344, 0.001) |
| *Lachnospiraceae_NK4A136_group* | 0.00791(0.00861, 0.00774) | 0.00219(0.05082, 0) | 0.00526(0.01046, 0) |
| *uncultured_bacterium_f_Lachnospiraceae* | 0.00511(0.00633, 0.00133) | 0.00872(0.0591,0.00088) | 0.00161(0.0028, 0) |
| *Clostridium_sensu_stricto_1* | 0 (0.00015, 0) | 0(0, 0) | 0.00412675(0.07843, 0.00017)**** |

Note: The relative abundances of the top 10 genera did not conform to a normal distribution and are shown as the median (upper-quartile, lower-quartile). Compare with HFDOP group, *****P* < 0.0001, n = 3.
